# Crystal structure of 2-(1,3-dioxoindan-2-yl)iso­quinoline-1,3,4-trione

**DOI:** 10.1107/S2056989014025997

**Published:** 2015-01-01

**Authors:** Raza Murad Ghalib, C. S. Chidan Kumar, Rokiah Hashim, Othman Sulaiman, Hoong-Kun Fun

**Affiliations:** a Department of Chemistry, Faculty of Sciences & Arts Khulais, King Abdulaziz University, Jeddah, KSA; b X-ray Crystallography Unit, School of Physics, Universiti Sains Malaysia, 11800 USM, Penang, Malaysia; c Department of Chemistry, Alva’s Institute of Engineering & Technology, Mijar, Moodbidri 574 225, Karnataka, India; d School of Industrial Technology, Universiti Sains Malaysia, 11800 USM, Penang, Malaysia; e Department of Pharmaceutical Chemistry, College of Pharmacy, King Saud University, PO Box 2457, Riaydh 11451, Saudi Arabia

**Keywords:** crystal structure, iso­quinoline-1,3,4-trione derivative, synthesis, hydrogen bonding, pharmacological properties

## Abstract

In the title iso­quinoline-1,3,4-trione derivative, C_18_H_9_NO_5_, the five-membered ring of the indane fragment adopts an envelope conformation with the nitro­gen-substituted C atom being the flap. The planes of the indane benzene ring and the iso­quinoline-1,3,4-trione ring make a dihedral angle of 82.06 (6)°. In the crystal, mol­ecules are linked into chains extending along the *bc* plane *via* C—H⋯O hydrogen-bonding inter­actions, enclosing *R*_2_^2^(8) and *R*_2_^2^(10) loops. The chains are further connected by π–π stacking inter­ations, with centroid-to-centroid distances of 3.9050 (7) Å, forming layers parallel to the *b* axis.

## Related literature   

For the biological activity of iso­quinoline-1,3,4-triones, see: Chen *et al.* (2006 ▸); Du *et al.* (2008 ▸). For related iso­quinoline-1,3,4-trione structures, see: Yu *et al.* (2010 ▸); Huang *et al.* (2013 ▸). For synthetic applications of iso­quinoline-1,3,4-trione, see: Yu *et al.* (2010 ▸); Huang *et al.* (2011 ▸, 2013 ▸). For the synthesis of related compounds, see: Chen *et al.* (2006 ▸); Du *et al.* (2008 ▸); Ghalib *et al.* 2011 ▸; Schaber *et al.* 2004 ▸; Huang *et al.* (2013 ▸).
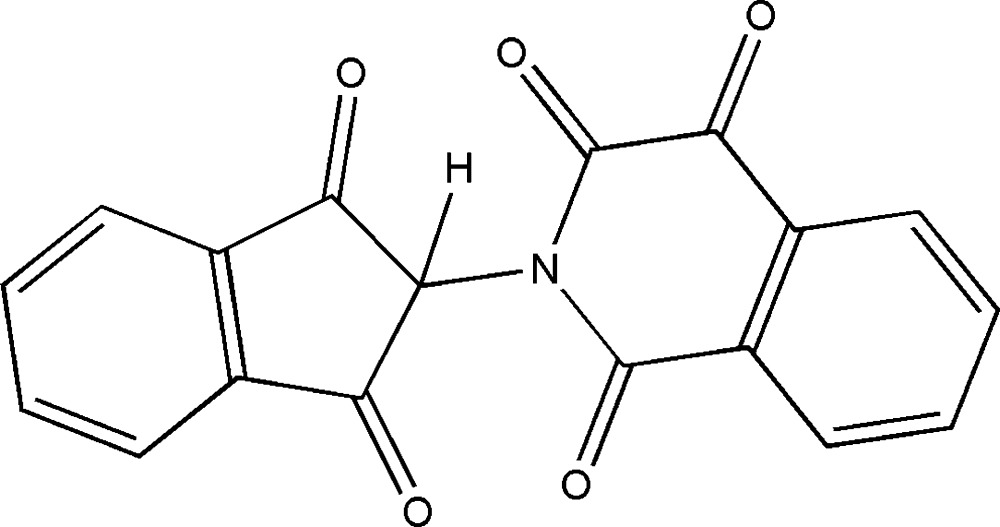


## Experimental   

### Crystal data  

C_18_H_9_NO_5_
*M* *_r_* = 319.26
Monoclinic, 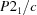

*a* = 12.6080 (1) Å
*b* = 13.6849 (2) Å
*c* = 8.4467 (1) Å
β = 102.051 (1)°
*V* = 1425.27 (3) Å^3^
*Z* = 4
Cu *K*α radiation
μ = 0.93 mm^−1^
*T* = 100 K
0.24 × 0.15 × 0.14 mm

### Data collection  

Bruker APEXII CCD diffractometer
Absorption correction: multi-scan (*SADABS*; Bruker, 2009 ▸) *T*_min_ = 0.808, *T*_max_ = 0.879
9639 measured reflections
2597 independent reflections
2458 reflections with *I* > 2σ(*I*)
*R*_int_ = 0.024

### Refinement  

*R*[*F* ^2^ > 2σ(*F* ^2^)] = 0.035
*wR*(*F* ^2^) = 0.093
*S* = 1.04
2597 reflections
217 parameters
H-atom parameters constrained
Δρ_max_ = 0.27 e Å^−3^
Δρ_min_ = −0.20 e Å^−3^

### 

Data collection: *APEX2* (Bruker, 2009 ▸); cell refinement: *SAINT* (Bruker, 2009 ▸); data reduction: *SAINT*; program(s) used to solve structure: *SHELXS97* (Sheldrick, 2008 ▸); program(s) used to refine structure: *SHELXL2013* (Sheldrick, 2008 ▸); molecular graphics: *SHELXTL* (Sheldrick, 2008 ▸); software used to prepare material for publication: *SHELXTL* and *PLATON* (Spek, 2009 ▸).

## Supplementary Material

Crystal structure: contains datablock(s) I, New_Global_Publ_Block. DOI: 10.1107/S2056989014025997/zl2607sup1.cif

Structure factors: contains datablock(s) I. DOI: 10.1107/S2056989014025997/zl2607Isup2.hkl

Supporting information file. DOI: 10.1107/S2056989014025997/zl2607Isup3.cml

**Fig. 1.**
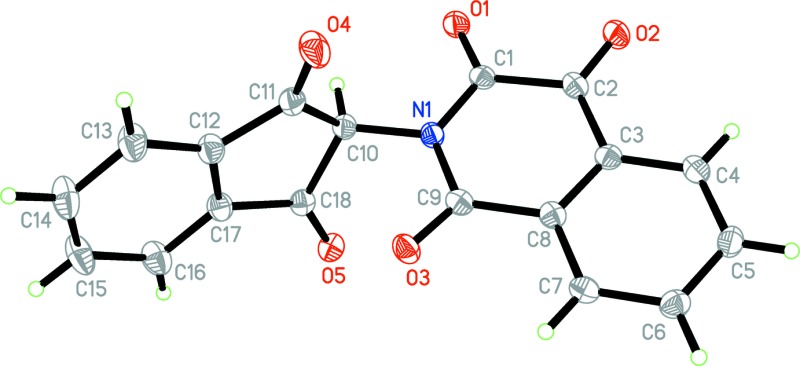
The molecular structure of the title compound with atom labels and 50% probability displacement ellipsoids.

. DOI: 10.1107/S2056989014025997/zl2607fig1.tif
The mol­ecular structure of the title compound with atom labels and 50% probability displacement ellipsoids.

**Fig. 2.**
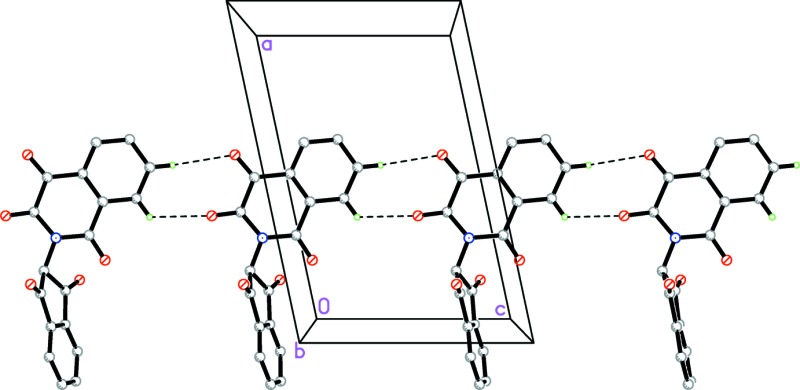
Crystal packing of the title compound, showing the C6–H6A···O2 and C7–H7A···O1 hydrogen bonding interactions (Symmetry codes: x, y, z + 1) as dashed lines incorporating R22(8) loops. Other H-atoms are omited for clarity.

Click here for additional data file.A A x y z . DOI: 10.1107/S2056989014025997/zl2607fig2.tif
Crystal packing of the title compound, showing the C6–H6*A*⋯O2 and C7–H7*A*⋯O1 hydrogen bonding inter­actions (Symmetry codes: *x*, *y*, *z* + 1) as dashed lines incorporating 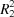
(8) loops. Other H-atoms are omited for clarity.

**Fig. 3.**
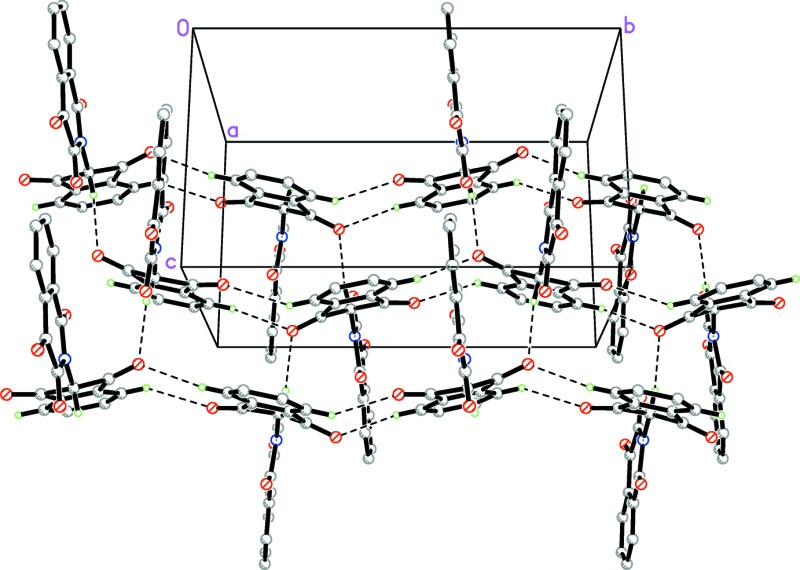
Crystal packing of the title compound, showing the C–H···O hydrogen bonding interactions (Symmetry codes: x, -y + 1/2, z - 1/2; -x, y + 1/2, -z - 1/2; -x, y - 1/2, -z - 1/2) as dashed lines incorporating R22(10) loops. Other H-atoms are omited for clarity.

Click here for additional data file.x y z x y z x y z . DOI: 10.1107/S2056989014025997/zl2607fig3.tif
Crystal packing of the title compound, showing the C–H⋯O hydrogen bonding inter­actions (Symmetry codes: *x*, −*y* + 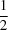, *z* − 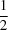; −*x*, *y* + 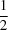, −*z* − 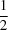; −*x*, *y* − 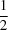, −*z* − 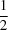) as dashed lines incorporating 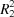
(10) loops. Other H-atoms are omited for clarity.

**Fig. 4.**
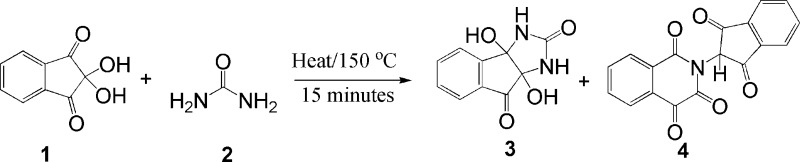
Reaction scheme for the title compound.

. DOI: 10.1107/S2056989014025997/zl2607fig4.tif
Reaction scheme for the title compound.

CCDC reference: 1036387

Additional supporting information:  crystallographic information; 3D view; checkCIF report

## Figures and Tables

**Table 1:** Hydrogen-bond geometry (, )

*D*H*A*	*D*H	H*A*	*D* *A*	*D*H*A*
C6H6*A*O2^i^	0.95	2.54	3.4862(16)	171
C7H7*A*O1^i^	0.95	2.51	3.1397(15)	124
C10H10*A*O5^ii^	1.00	2.24	3.2022(15)	161
C13H13*A*O5^iii^	0.95	2.37	3.2852(16)	163
C16H16*A*O4^iv^	0.95	2.50	3.3596(17)	150

Symmetry codes: (i) 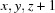; (ii) 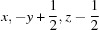; (iii) 
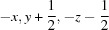
; (iv) 
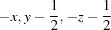
.

## supplementary crystallographic information

### S1. Chemical context

Urea has a complicated thermal behavior. It is thermally very liable to change. Thermal decomposition of urea under open reaction vessel conditions at temperatures in excess of 152 °C primarily gives cyanic acid (HNCO). HNCO on contact with additional urea in turn yields biuret which at a temperature of greater than 190 °C is liable to transform into cyanuric acid (Schaber *et al.*, 2004). High-temperature thermal decomposition of cyanuric acid also gives cyanic acid again.

Heating of a mixture of ninhydrin and urea above the melting point of urea gives a mixture of 3a,8a-di­hydroxy-1,3,3a,8a-tetra­hydro- indeno­[1,2-d]imidazole-2,8-dione (3) and the title compound 2-(1,3-dioxoindan-2-yl)-iso­quinoline-1,3,4-trione (4) in about equal amounts, figure 4 (Ghalib *et al.* 2011). Compound 4 is most probably the product of reaction of nihyrin with cyanic acid. The formation of an iso­quinoline-1,3,4-triones is of inter­est as some of these compounds have been known for their potent anti­cancer activity (Chen *et al.*, 2006; Du *et al.* 2008). In continuation to our inter­est in the chemical and pharmacological properties of ninhydrin derivatives (Ghalib *et al.*, 2011), we synthesized the title compound 4 as a precursor for the synthesis of potential chemotherapeutic agents (Chen *et al.*, 2006).

### S2. Structural commentary

In the title compound (Fig. 1), the study of torsion angles, asymmetric parameters and least squares planes reveals that the indane (C10–C12/C17/C18) ring adopts an envelope conformation with the nitro­gen substituted C atom deviating by -0.104 (1) Å from the least-squares plane. The indane benzene ring (C12–C17) and the iso­quinoline-1,3,4-trione ring exhibit a dihedral angle of 82.06 (6)°, suggesting they are almost perpendicular to each other.

### S3. Supra­molecular features

In the crystal structure, the molecules are connected into chains extending along the *bc* plane *via* inter­molecular C–H···O hydrogen bonds (Table 1) enclosing R_2_^2^(8) and R_2_^2^(10) loops (Fig. 2 & 3). In addition, π–π inter­actions (*C*g2···*C*g3 = 3.9050 (7) Å; symmetry code: 1-x, 1-y, 1-z) stack the molecules into layers parallel to the *b* axis, where *C*g2 and *C*g3 are the centroids of the pyridine-2,3,6-trione and the benzene (C3–C8) rings respectively.

### S4. Synthesis and crystallization

A dry mixture of ninhydrin (1) (1.78 g) and urea (2) (0.60 g) in molar ratio 1:1 was heated for 15 minutes to 150 °C above the melting point of urea (130–135 °C). The reaction mixture was cooled and then fractionally crystallized with an alcohol-chloro­form (1:1) mixture to give colorless crystals of 3 as 3a,8a-di­hydroxy-1,3,3a,8a-tetra­hydro- indeno­[1,2-d]imidazole-2,8-dione (yield 40%, M.P.: 220 °C) (Ghalib *et al.* 2011) and brownish crystals of the title compound 4 as 2-(1,3-dioxo-indan-2-yl)-iso­quinoline-1,3,4-trione (yield 35%, m.p., 290 °C, Fig. 4).

### S5. Refinement details

All the H atoms were positioned geometrically (C=H 0.93–0.98 Å) and refined using a riding model with *U*_iso_(H) = 1.2 *U*_eq_(C).

### Crystal data

**Table:**

C_18_H_9_NO_5_	*F*(000) = 656
*M**_r_* = 319.26	*D*_x_ = 1.488 Mg m^−^^3^
Monoclinic, *P*2_1_/*c*	Cu *K*α radiation, λ = 1.54178 Å
*a* = 12.6080 (1) Å	Cell parameters from 6347 reflections
*b* = 13.6849 (2) Å	θ = 6.3–71.7°
*c* = 8.4467 (1) Å	µ = 0.93 mm^−^^1^
β = 102.051 (1)°	*T* = 100 K
*V* = 1425.27 (3) Å^3^	Block, orange
*Z* = 4	0.24 × 0.15 × 0.14 mm

### Data collection

**Table:**

Bruker APEXII CCD diffractometer	2458 reflections with *I* > 2σ(*I*)
Radiation source: fine-focus sealed tube	*R*_int_ = 0.024
φ and ω scans	θ_max_ = 72.0°, θ_min_ = 6.3°
Absorption correction: multi-scan (*SADABS*; Bruker, 2009)	*h* = −15→14
*T*_min_ = 0.808, *T*_max_ = 0.879	*k* = −16→16
9639 measured reflections	*l* = −9→8
2597 independent reflections	

### Refinement

**Table:**

Refinement on *F*^2^	0 restraints
Least-squares matrix: full	Hydrogen site location: inferred from neighbouring sites
*R*[*F*^2^ > 2σ(*F*^2^)] = 0.035	H-atom parameters constrained
*wR*(*F*^2^) = 0.093	*w* = 1/[σ^2^(*F*_o_^2^) + (0.0504*P*)^2^ + 0.5485*P*] where *P* = (*F*_o_^2^ + 2*F*_c_^2^)/3
*S* = 1.04	(Δ/σ)_max_ < 0.001
2597 reflections	Δρ_max_ = 0.27 e Å^−^^3^
217 parameters	Δρ_min_ = −0.20 e Å^−^^3^

### Special details

**Table:**

Geometry. All e.s.d.'s (except the e.s.d. in the dihedral angle between two l.s. planes) are estimated using the full covariance matrix. The cell e.s.d.'s are taken into account individually in the estimation of e.s.d.'s in distances, angles and torsion angles; correlations between e.s.d.'s in cell parameters are only used when they are defined by crystal symmetry. An approximate (isotropic) treatment of cell e.s.d.'s is used for estimating e.s.d.'s involving l.s. planes.

### Fractional atomic coordinates and isotropic or equivalent isotropic displacement parameters (Å^2^)

**Table:**

	*x*	*y*	*z*	*U*_iso_*/*U*_eq_	
O1	0.36681 (7)	0.34686 (7)	−0.36897 (11)	0.0226 (2)	
O2	0.56844 (7)	0.37737 (7)	−0.18918 (11)	0.0244 (2)	
O3	0.22076 (7)	0.37449 (6)	0.07501 (11)	0.0212 (2)	
O4	0.14773 (7)	0.52030 (7)	−0.25876 (13)	0.0310 (3)	
O5	0.14677 (7)	0.18880 (6)	−0.12358 (10)	0.0208 (2)	
N1	0.29330 (8)	0.35524 (7)	−0.14517 (12)	0.0167 (2)	
C1	0.37928 (9)	0.35678 (8)	−0.22453 (15)	0.0171 (3)	
C2	0.49378 (10)	0.37240 (8)	−0.11979 (15)	0.0182 (3)	
C3	0.50578 (10)	0.38012 (8)	0.05673 (15)	0.0173 (3)	
C4	0.60836 (10)	0.39054 (9)	0.15659 (16)	0.0202 (3)	
H4A	0.6710	0.3933	0.1108	0.024*	
C5	0.61838 (10)	0.39680 (9)	0.32253 (16)	0.0219 (3)	
H5A	0.6880	0.4043	0.3907	0.026*	
C6	0.52631 (10)	0.39207 (9)	0.39026 (16)	0.0213 (3)	
H6A	0.5339	0.3947	0.5045	0.026*	
C7	0.42389 (10)	0.38360 (8)	0.29149 (15)	0.0191 (3)	
H7A	0.3614	0.3817	0.3377	0.023*	
C8	0.41329 (10)	0.37786 (8)	0.12429 (15)	0.0172 (3)	
C9	0.30286 (10)	0.36955 (8)	0.02176 (15)	0.0172 (3)	
C10	0.18389 (9)	0.34521 (9)	−0.24087 (15)	0.0180 (3)	
H10A	0.1894	0.3278	−0.3539	0.022*	
C11	0.11456 (10)	0.43817 (9)	−0.24898 (16)	0.0210 (3)	
C12	0.00327 (10)	0.40620 (9)	−0.24322 (16)	0.0215 (3)	
C13	−0.09105 (10)	0.46146 (10)	−0.26425 (18)	0.0276 (3)	
H13A	−0.0910	0.5291	−0.2893	0.033*	
C14	−0.18531 (11)	0.41422 (10)	−0.2473 (2)	0.0314 (3)	
H14A	−0.2510	0.4502	−0.2610	0.038*	
C15	−0.18544 (11)	0.31468 (10)	−0.21033 (19)	0.0315 (3)	
H15A	−0.2513	0.2842	−0.2000	0.038*	
C16	−0.09097 (11)	0.25943 (10)	−0.18845 (18)	0.0267 (3)	
H16A	−0.0910	0.1918	−0.1629	0.032*	
C17	0.00336 (10)	0.30685 (9)	−0.20540 (15)	0.0201 (3)	
C18	0.11462 (9)	0.26700 (8)	−0.18079 (14)	0.0173 (3)	

### Atomic displacement parameters (Å^2^)

**Table:**

	*U*^11^	*U*^22^	*U*^33^	*U*^12^	*U*^13^	*U*^23^
O1	0.0203 (4)	0.0298 (5)	0.0183 (5)	−0.0022 (3)	0.0056 (3)	−0.0011 (4)
O2	0.0176 (4)	0.0350 (5)	0.0221 (5)	−0.0004 (3)	0.0075 (4)	0.0005 (4)
O3	0.0169 (4)	0.0267 (5)	0.0214 (5)	−0.0016 (3)	0.0072 (3)	−0.0031 (3)
O4	0.0215 (5)	0.0205 (5)	0.0501 (7)	−0.0025 (4)	0.0055 (4)	0.0081 (4)
O5	0.0218 (4)	0.0175 (4)	0.0219 (5)	0.0011 (3)	0.0019 (3)	−0.0010 (3)
N1	0.0137 (5)	0.0192 (5)	0.0172 (5)	−0.0005 (4)	0.0031 (4)	−0.0007 (4)
C1	0.0180 (6)	0.0152 (6)	0.0189 (7)	0.0001 (4)	0.0055 (5)	0.0008 (4)
C2	0.0169 (6)	0.0160 (6)	0.0222 (7)	0.0003 (4)	0.0055 (5)	0.0006 (5)
C3	0.0180 (6)	0.0149 (5)	0.0190 (6)	0.0000 (4)	0.0043 (5)	0.0012 (4)
C4	0.0180 (6)	0.0196 (6)	0.0234 (7)	−0.0004 (4)	0.0054 (5)	0.0014 (5)
C5	0.0185 (6)	0.0224 (6)	0.0226 (7)	−0.0017 (5)	−0.0004 (5)	0.0007 (5)
C6	0.0250 (6)	0.0207 (6)	0.0180 (6)	−0.0017 (5)	0.0036 (5)	−0.0004 (5)
C7	0.0206 (6)	0.0178 (6)	0.0202 (6)	−0.0010 (4)	0.0069 (5)	−0.0003 (4)
C8	0.0181 (6)	0.0138 (5)	0.0198 (6)	−0.0005 (4)	0.0039 (5)	0.0002 (4)
C9	0.0180 (6)	0.0146 (5)	0.0198 (6)	−0.0006 (4)	0.0060 (5)	−0.0006 (4)
C10	0.0157 (6)	0.0203 (6)	0.0174 (6)	−0.0002 (4)	0.0024 (4)	0.0001 (4)
C11	0.0177 (6)	0.0207 (6)	0.0237 (7)	−0.0003 (5)	0.0022 (5)	0.0039 (5)
C12	0.0178 (6)	0.0202 (6)	0.0257 (7)	−0.0012 (5)	0.0031 (5)	0.0000 (5)
C13	0.0203 (6)	0.0192 (6)	0.0423 (8)	0.0017 (5)	0.0042 (5)	0.0014 (6)
C14	0.0177 (6)	0.0261 (7)	0.0497 (9)	0.0029 (5)	0.0058 (6)	−0.0034 (6)
C15	0.0188 (6)	0.0264 (7)	0.0508 (9)	−0.0049 (5)	0.0107 (6)	−0.0036 (6)
C16	0.0222 (6)	0.0189 (6)	0.0398 (8)	−0.0028 (5)	0.0083 (5)	−0.0010 (5)
C17	0.0183 (6)	0.0195 (6)	0.0225 (7)	−0.0007 (5)	0.0039 (5)	−0.0019 (5)
C18	0.0177 (6)	0.0180 (6)	0.0157 (6)	−0.0019 (4)	0.0025 (4)	−0.0035 (4)

### Geometric parameters (Å, º)

**Table:**

O1—C1	1.2048 (15)	C7—C8	1.3927 (18)
O2—C2	1.2102 (15)	C7—H7A	0.9500
O3—C9	1.2136 (15)	C8—C9	1.4825 (17)
O4—C11	1.2080 (16)	C10—C18	1.5332 (16)
O5—C18	1.2088 (15)	C10—C11	1.5370 (16)
N1—C1	1.3886 (15)	C10—H10A	1.0000
N1—C9	1.4036 (16)	C11—C12	1.4802 (17)
N1—C10	1.4525 (15)	C12—C13	1.3890 (18)
C1—C2	1.5424 (16)	C12—C17	1.3966 (17)
C2—C3	1.4707 (17)	C13—C14	1.3863 (19)
C3—C4	1.3961 (17)	C13—H13A	0.9500
C3—C8	1.4016 (17)	C14—C15	1.398 (2)
C4—C5	1.3835 (18)	C14—H14A	0.9500
C4—H4A	0.9500	C15—C16	1.3901 (19)
C5—C6	1.3987 (18)	C15—H15A	0.9500
C5—H5A	0.9500	C16—C17	1.3883 (18)
C6—C7	1.3881 (18)	C16—H16A	0.9500
C6—H6A	0.9500	C17—C18	1.4787 (16)
			
C1—N1—C9	124.81 (10)	N1—C10—C18	114.97 (10)
C1—N1—C10	118.64 (10)	N1—C10—C11	114.32 (10)
C9—N1—C10	116.40 (10)	C18—C10—C11	103.57 (9)
O1—C1—N1	122.48 (11)	N1—C10—H10A	107.9
O1—C1—C2	120.34 (11)	C18—C10—H10A	107.9
N1—C1—C2	117.17 (10)	C11—C10—H10A	107.9
O2—C2—C3	124.14 (11)	O4—C11—C12	128.41 (12)
O2—C2—C1	117.35 (11)	O4—C11—C10	124.84 (11)
C3—C2—C1	118.51 (10)	C12—C11—C10	106.74 (10)
C4—C3—C8	120.06 (11)	C13—C12—C17	121.27 (12)
C4—C3—C2	120.40 (11)	C13—C12—C11	128.83 (12)
C8—C3—C2	119.54 (11)	C17—C12—C11	109.86 (11)
C5—C4—C3	119.71 (11)	C14—C13—C12	117.55 (12)
C5—C4—H4A	120.1	C14—C13—H13A	121.2
C3—C4—H4A	120.1	C12—C13—H13A	121.2
C4—C5—C6	120.23 (11)	C13—C14—C15	121.24 (12)
C4—C5—H5A	119.9	C13—C14—H14A	119.4
C6—C5—H5A	119.9	C15—C14—H14A	119.4
C7—C6—C5	120.36 (12)	C16—C15—C14	121.26 (12)
C7—C6—H6A	119.8	C16—C15—H15A	119.4
C5—C6—H6A	119.8	C14—C15—H15A	119.4
C6—C7—C8	119.63 (11)	C17—C16—C15	117.44 (12)
C6—C7—H7A	120.2	C17—C16—H16A	121.3
C8—C7—H7A	120.2	C15—C16—H16A	121.3
C7—C8—C3	119.98 (11)	C16—C17—C12	121.25 (11)
C7—C8—C9	118.45 (11)	C16—C17—C18	128.40 (11)
C3—C8—C9	121.57 (11)	C12—C17—C18	110.28 (10)
O3—C9—N1	118.63 (11)	O5—C18—C17	127.71 (11)
O3—C9—C8	123.28 (11)	O5—C18—C10	125.71 (11)
N1—C9—C8	118.09 (10)	C17—C18—C10	106.57 (10)
			
C9—N1—C1—O1	−177.84 (11)	C1—N1—C10—C18	131.20 (11)
C10—N1—C1—O1	−2.54 (16)	C9—N1—C10—C18	−53.10 (13)
C9—N1—C1—C2	1.89 (16)	C1—N1—C10—C11	−109.16 (12)
C10—N1—C1—C2	177.19 (9)	C9—N1—C10—C11	66.53 (13)
O1—C1—C2—O2	2.38 (17)	N1—C10—C11—O4	38.25 (18)
N1—C1—C2—O2	−177.36 (10)	C18—C10—C11—O4	164.10 (13)
O1—C1—C2—C3	−177.50 (11)	N1—C10—C11—C12	−142.00 (11)
N1—C1—C2—C3	2.76 (15)	C18—C10—C11—C12	−16.15 (13)
O2—C2—C3—C4	−2.32 (18)	O4—C11—C12—C13	7.3 (2)
C1—C2—C3—C4	177.55 (10)	C10—C11—C12—C13	−172.42 (13)
O2—C2—C3—C8	177.08 (11)	O4—C11—C12—C17	−170.35 (14)
C1—C2—C3—C8	−3.05 (16)	C10—C11—C12—C17	9.92 (14)
C8—C3—C4—C5	1.19 (17)	C17—C12—C13—C14	−0.4 (2)
C2—C3—C4—C5	−179.41 (11)	C11—C12—C13—C14	−177.81 (14)
C3—C4—C5—C6	0.42 (18)	C12—C13—C14—C15	0.0 (2)
C4—C5—C6—C7	−1.67 (19)	C13—C14—C15—C16	0.3 (2)
C5—C6—C7—C8	1.27 (18)	C14—C15—C16—C17	−0.3 (2)
C6—C7—C8—C3	0.35 (17)	C15—C16—C17—C12	−0.1 (2)
C6—C7—C8—C9	−179.58 (10)	C15—C16—C17—C18	176.66 (13)
C4—C3—C8—C7	−1.59 (17)	C13—C12—C17—C16	0.4 (2)
C2—C3—C8—C7	179.01 (10)	C11—C12—C17—C16	178.31 (12)
C4—C3—C8—C9	178.35 (10)	C13—C12—C17—C18	−176.86 (12)
C2—C3—C8—C9	−1.06 (16)	C11—C12—C17—C18	1.01 (15)
C1—N1—C9—O3	174.03 (10)	C16—C17—C18—O5	−9.2 (2)
C10—N1—C9—O3	−1.36 (15)	C12—C17—C18—O5	167.87 (12)
C1—N1—C9—C8	−5.98 (16)	C16—C17—C18—C10	171.40 (13)
C10—N1—C9—C8	178.63 (10)	C12—C17—C18—C10	−11.55 (14)
C7—C8—C9—O3	5.45 (17)	N1—C10—C18—O5	−37.31 (17)
C3—C8—C9—O3	−174.48 (11)	C11—C10—C18—O5	−162.74 (12)
C7—C8—C9—N1	−174.54 (10)	N1—C10—C18—C17	142.12 (10)
C3—C8—C9—N1	5.52 (16)	C11—C10—C18—C17	16.70 (12)

### Hydrogen-bond geometry (Å, º)

**Table:**

*D*—H···*A*	*D*—H	H···*A*	*D*···*A*	*D*—H···*A*
C6—H6*A*···O2^i^	0.95	2.54	3.4862 (16)	171
C7—H7*A*···O1^i^	0.95	2.51	3.1397 (15)	124
C10—H10*A*···O5^ii^	1.00	2.24	3.2022 (15)	161
C13—H13*A*···O5^iii^	0.95	2.37	3.2852 (16)	163
C16—H16*A*···O4^iv^	0.95	2.50	3.3596 (17)	150

Symmetry codes: (i) *x*, *y*, *z*+1; (ii) *x*, −*y*+1/2, *z*−1/2; (iii) −*x*, *y*+1/2, −*z*−1/2; (iv) −*x*, *y*−1/2, −*z*−1/2.
