# Supplementary figures and images for: Crystal structure of 2-(1,3-dioxoindan-2-yl)iso­quinoline-1,3,4-trione
Source: Acta Crystallogr E Crystallogr Commun. 2015 Jan 1;71(Pt 1):o6–7. doi: 10.1107/S2056989014025997 (PMC4331915; doi:10.1107/S2056989014025997)

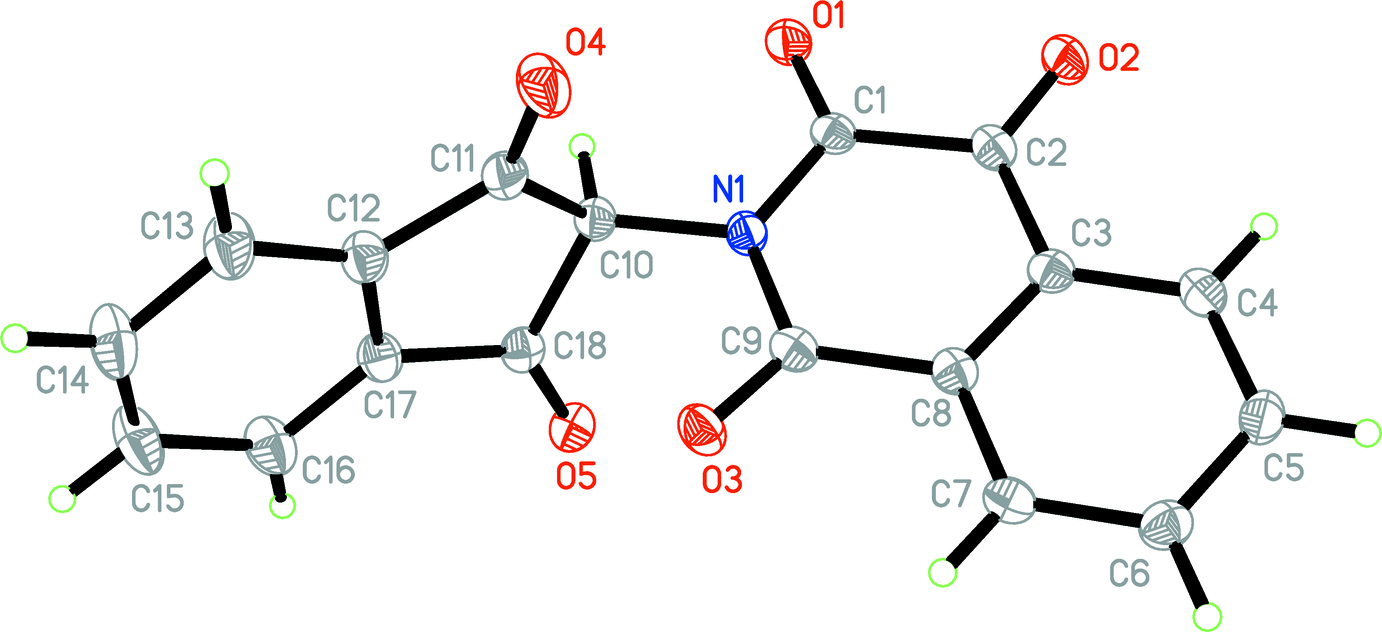

Supplement: Supplementary file 4 [file e-71-000o6-fig1.tif]

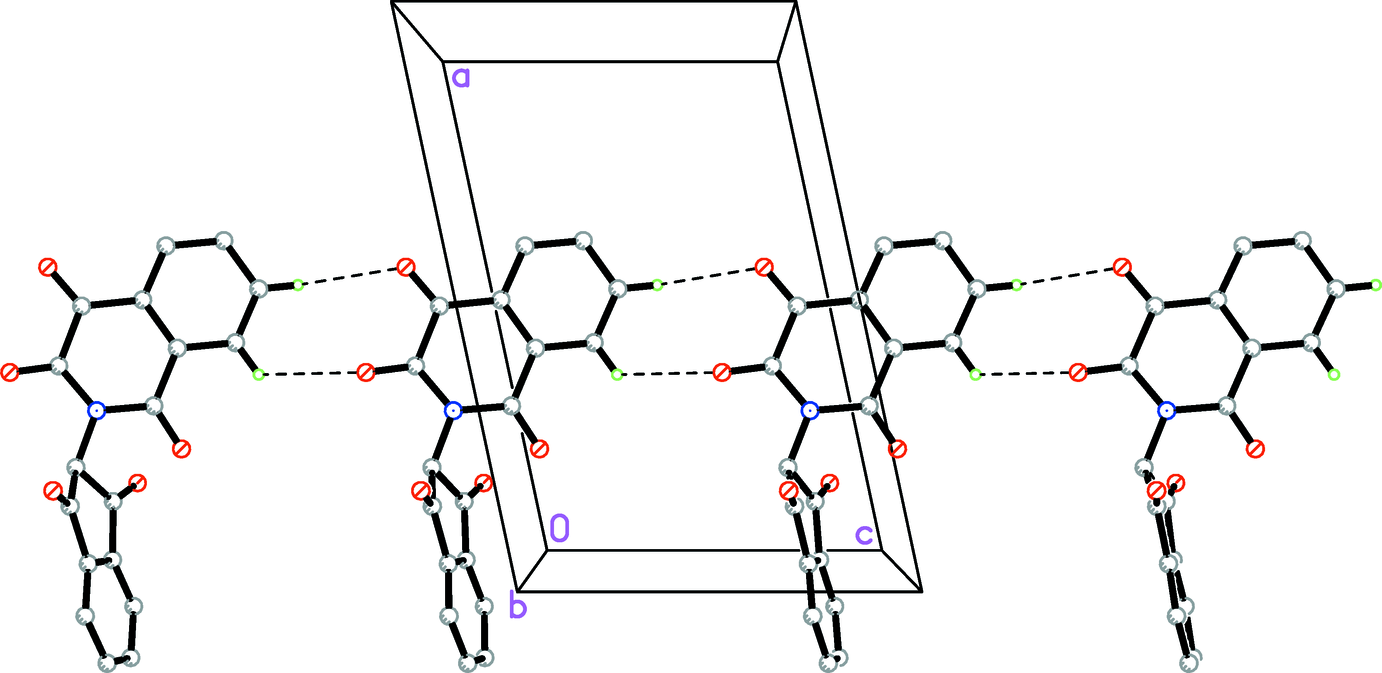

Supplement: Supplementary file 5 [file e-71-000o6-fig2.tif]

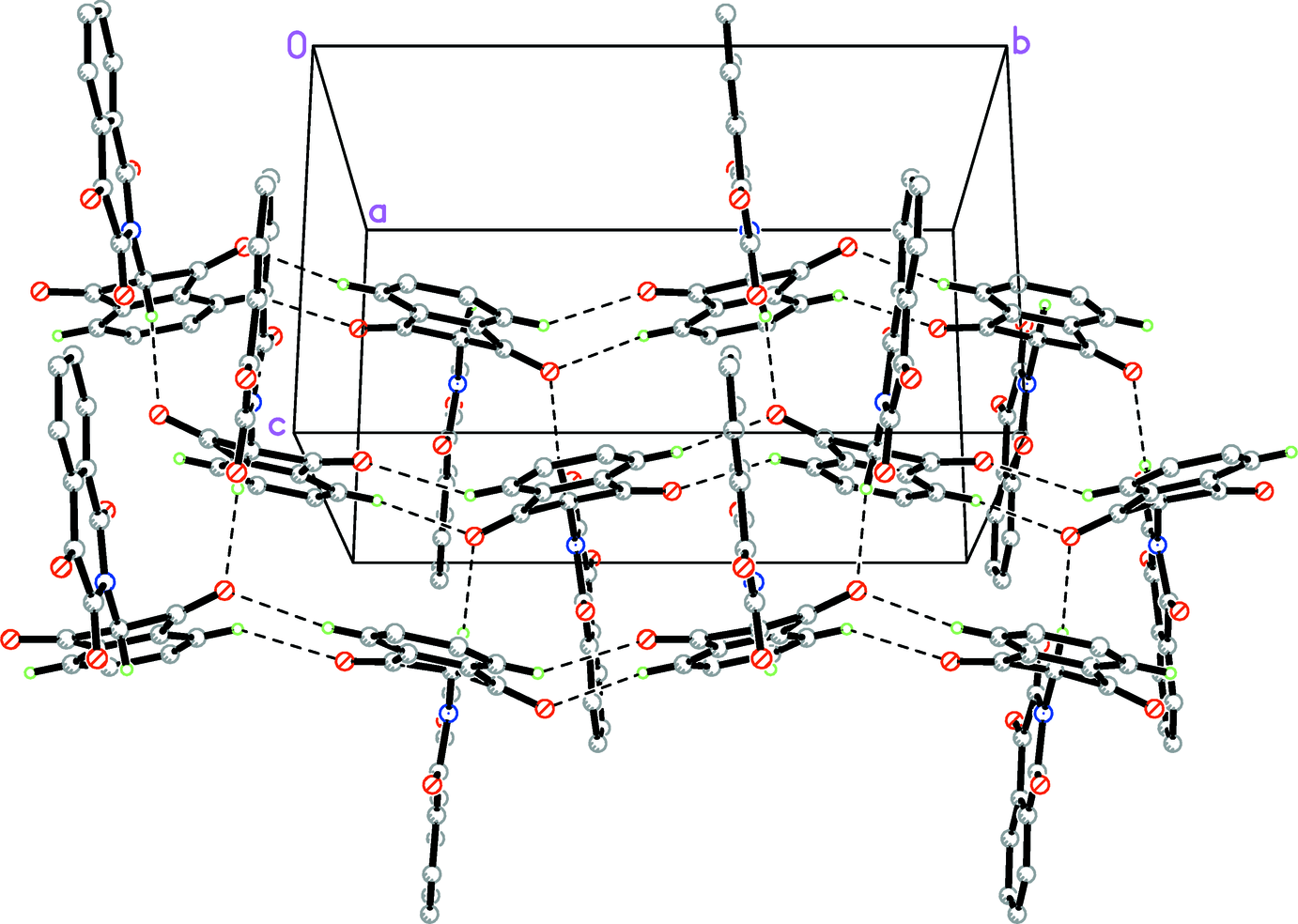

Supplement: Supplementary file 6 [file e-71-000o6-fig3.tif]
